# Ferrocene-Modified Block Copolymers for the Preparation of Smart Porous Membranes

**DOI:** 10.3390/polym9100491

**Published:** 2017-10-08

**Authors:** Sebastian Schöttner, Rimjhim Hossain, Christian Rüttiger, Markus Gallei

**Affiliations:** Ernst-Berl Institute for Chemical Engineering and Macromolecular Science, Technische Universität Darmstadt, Alarich-Weiss-Str. 4, D-64287 Darmstadt, Germany; s.schoettner@mc.tu-darmstadt.de (S.S.); rimjhimhossain@yahoo.de (R.H.); c.ruettiger@mc.tu-darmstadt.de (C.R.)

**Keywords:** block copolymers, metallopolymers, self-assembly, postmodification, membranes, stimuli-responsive polymers, phase inversion

## Abstract

The design of artificially generated channels featuring distinct remote-switchable functionalities is of critical importance for separation, transport control, and water filtration applications. Here, we focus on the preparation of block copolymers (BCPs) consisting of polystyrene-*block*-poly(2-hydroxyethyl methacrylate) (PS-*b*-PHEMA) having molar masses in the range of 91 to 124 kg mol^−1^ with a PHEMA content of 13 to 21 mol %. The BCPs can be conveniently functionalized with redox-active ferrocene moieties by a postmodification protocol for the hydrophilic PHEMA segments. Up to 66 mol % of the hydroxyl functionalities can be efficiently modified with the reversibly redox-responsive units. For the first time, the ferrocene-containing BCPs are shown to form nanoporous integral asymmetric membranes by self-assembly and application of the non-solvent-induced phase separation (SNIPS) process. Open porous structures are evidenced by scanning electron microscopy (SEM) and water flux measurements, while efficient redox-switching capabilities are investigated after chemical oxidation of the ferrocene moieties. As a result, the porous membranes reveal a tremendously increased polarity after oxidation as reflected by contact angle measurements. Additionally, the initial water flux of the ferrocene-containing membranes decreased after oxidizing the ferrocene moieties because of oxidation-induced pore swelling of the membrane.

## 1. Introduction

Stimuli-responsive membranes attract enormous attention because of their excellent control over permeability, selectivity, and absorption capabilities stimulated by external triggers [1,2,3,4]. The elaborated design of synthetic channels resulting in transport control is of critical importance for instance in separation and filtration applications. In general, stimuli-responsive polymer materials attracted enormous attention for a range of recent polymer-based applications [5,6,7]. This type of polymers feature certain chemical functionalities, which can be addressed by external triggers such as the presence of solvent, change of temperature, variation of pH, by light, redox reagents, or electrical or magnetic fields [8,9,10,11]. Especially thermo-responsive membranes were shown to reversibly switch their pore diameter and surface properties, and therefore, controlling the selectivity of the membrane [4,12,13,14,15,16]. In general, among the broad variety of stimuli-responsive mechanisms, redox reactions induced by the addition of oxidation or reducing agents or by applying electrical potentials are much less investigated. In the recent past, the ferrocene/ferrocenium redox couple in metallopolymers has attracted significant interest due to the unique capability of (electro)chemical switching between hydrophobic ferrocene and comparably hydrophilic ferrocenium moieties [17,18,19,20,21,22,23,24]. In the field of (nano)porous structures, ionic permeation across smart membranes have been modulated by introducing redox-active moieties or polymers at the inner pore walls of the membrane [25,26,27,28]. Elbert et al. reported on the modification of mesoporous silica membranes with redox-responsive ferrocene-containing polymers by a grafting to and a grafting from approach [29]. Within this approach, successful redox-mediated ion permselectivity in mesoporous membranes was shown. As another example, Vancso et al. took advantage of redox-switchable poly(ferrocenylsilanes) (PFS) in polyelectrolyte multilayer capsules for changing the permeability [30]. In general, one major drawback of the porous membrane postmodification route is the subsequent (at least one) synthetic step for introducing the redox-active moieties. Moreover, if redox-active monomers were used for in-situ pore modification by polymerization, determination of constitution and molar mass of the immobilized polymers is coming with issues because of the low amount of attached polymer and possible undesirable side reactions during the postmodification. This may further influence the membrane performance. Therefore, the one-step preparation of porous membrane formation based on well-defined and tailored redox-responsive polymers would be advantageous. An elegant approach was reported by the Vancso group [31]: a porous polyelectrolyte membrane from PFS-based poly(ionic liquid)s and poly(acrylic acid) could be prepared. The stimuli-responsive characteristics were shown and the membrane was capable of reversibly changing the pore size after exposure to redox stimulation [31,32]. In general, for industrially relevant applications as asymmetric copolymer membranes the so-called self-assembly and non-solvent induced phase separation (SNIPS) process is of utmost importance [33]. Thereby, a concentrated polymer solution is cast on a macroporous support and immersed in a non-solvent bath. The solvent-non-solvent exchange leads to phase separation forming a selective layer on the top of the polymer film. In the last decade, functional block copolymer filtration membranes attracted enormous attention because of their isoporous surface structure and capability for responsiveness stimulated by external triggers [34,35,36,37,38,39,40,41]. The most prominent and first block copolymer in this field is polystyrene-*block*-poly(4-vinylpyridine) (PS-*b*-P4VP), which revealed tremendous potential for water purification and separation processes [42,43,44,45,46]. Quite recently, we reported on the preparation of the amphiphilic block copolymer polystyrene-*block*-poly(2-hydroxyethyl methacrylate) (PS-*b*-PHEMA) for the formation of integral asymmetric porous membranes featuring a high water flux [47]. Moreover, because of the hydroxyl-groups of the inner pore walls, sol-gel chemistry could be applied for converting these membranes into porous TiO_2_ while maintaining the pristine membrane structure.

Within the present study, PS-*b*-PHEMA block copolymers are modified with different amounts of ferrocene acid chloride, enabled by the well-established chemistry of the hydroxyl-groups of the PHEMA block segment. Structural and thermal characterization of the functionalized diblock copolymers is carried out involving NMR analysis, size exclusion chromatography (SEC), and differential scanning calorimetry (DSC). For the first time, the redox-active amphiphilic polymers are subjected to the SNIPS process for membrane formation. The novel integral asymmetric redox membranes are investigated with respect to structure formation, redox-switching capabilities, and water flux measurements.

## 2. Materials and Methods

### 2.1. Instrumentation

Standard SEC was performed with a system composed of a 1260 IsoPump-G1310B (Agilent Technologies, Santa Clara, CA, USA), a 1260 VW-detector-G1314F at 254 nm (Agilent Technologies) and a 1260 RI-detector-G1362A at 30 or 50 °C (Agilent Technologies), with THF (1 mg/mL) or DMF/LiCl (3 mg/mL) as mobile phase (flow rate 1 mL·min^−1^) on a SDV column set from PSS (from Polymer Standard Service, Mainz, Germany) (SDV 10^3^, SDV 10^5^, SDV 10^6^) or GRAM column set from PSS (GRAM 30, GRAM 1000, GRAM 1000). Calibration was carried out using PS (from Polymer Standard Service, Mainz, Germany) or PMMA standards. For data acquisition and evaluation, PSS WinGPC^®^ UniChrom 8.2 was used (from Polymer Standard Service, Mainz, Germany). NMR spectra were recorded with a Bruker DRX 500 NMR or with a Bruker DRX 300 spectrometer (Billerica, MA, USA) working at 500 or 300 MHz (^1^H NMR). NMR chemical shifts are referenced relative to tetramethylsilane or the used solvent. For determining the thermal properties of the materials, TGA was applied using a Mettler Toledo TGA 2 (Columbus, OH, USA) with a heating rate of 10 K·min^−1^ in the range of 30 to 600 °C in nitrogen atmosphere. Differential scanning calorimeter (DSC) was performed with a Mettler Toledo DSC-1 with a heating rate of 10 K·min^−1^ in a nitrogen atmosphere. Cyclic voltammetry (CV) measurements were carried out with a multipotentiostat VMP2 (Princeton Applied Research, Ametek, Oak Ridge, TN, USA) using EC-lab V10.12 software to collect the data. All measurements were carried out using an Ag/AgCl reference electrode, a Pt counter electrode, and a glassy carbon working electrode. The scan rate was 20 mV·s^−1^ in a range of −0.3 to 0.8 V for oxidation and reduction experiments. A 50 mL five-necked round-bottom flask was used as measurement cell with a tetrabutylammonium tetrafluoroborate (TBABF_4_) solution in degassed acetonitrile (0.1 M) as electrolyte and ferrocene as calibration. For Scanning electron microscopy (SEM) and energy-dispersive X-ray spectroscopy (EDS) a FEI/Philips XL30 FEG (Philips, Amsterdam, The Netherlands) with accelerating voltages between 5 and 30 kV was used. The SEM samples were coated with gold for 100 s at 30 mA using a Quorum Q300T D sputter coater (Lewes, UK). Transmission electron microscopy (TEM) experiments were carried out on a Zeiss EM 10 (Oberkochen, Germany) electron microscope operating at 60 kV. All shown images were recorded with a slow-scan CCD camera obtained from TRS (Tröndle TRS, Moorenweis, Germany) in bright field mode. Camera control was computer-aided using the ImageSP software from TRS (Tröndle TRS, Moorenweis, Germany). The contact angle (CA) was measured using the sessile-drop method with a Contact Angle System Data Physics OCA 15 EC using 2 μL droplets of deionized water. The measurements were conducted in a controlled climatic chamber at T = 23 °C ± 2 °C and a relative humidity of 40%. CAs were determined geometrically using the SCA20 software (DataPhysics Instruments GmbH, Filderstadt, Germany) by aligning a tangent from the surficial contact point along the droplets surface in the droplet profile. Water flux measurements were performed using a Merck Amicon stirred cell (Model 8010, 10 mL, Merck, Darmstadt, Germany) at a constant pressure of 1.0 bar using Millipore water (conductivity: 0.054 mS). The cell featured a filtration area of 4.1 cm^2^, and the membrane diameter was 2.5 cm.

### 2.2. Materials

All solvents and reagents were purchased from Alfa Aesar (Haverhill, MA, USA), Sigma-Aldrich (St. Louis, MA, USA), Fisher Scientific (Hampton, NH, USA), and ABCR (Karlsruhe, Germany) and used as received unless otherwise described. Deuterated solvents were purchased from Deutero GmbH (Kastellaun, Germany) or Sigma-Aldrich. Tetrahydrofuran (THF) was distilled from sodium/benzophenone under reduced pressure (cryo-transfer) prior to the addition of 1,1-diphenylethylene (DPE) and *n*-butyllithium (*n*-BuLi) followed by a second cryo-transfer. Prior to use for the anionic polymerization, the monomers styrene and 2-(Trimethylsilyloxy)ethyl methacrylate (HEMA-TMS) were distilled over calcium hydride (CaH_2_) and trioctylaluminum (25 wt % solution in hexane) and stored in a glovebox at −18 °C. Lithium chloride (LiCl) was suspended in freshly distilled THF and treated with *sec*-butyllithium (*s*-BuLi). Then, THF was removed in vacuo, and the dried LiCl was stored in a glovebox. All syntheses were carried out under an atmosphere of nitrogen using Schlenk techniques or a glovebox equipped with a Coldwell apparatus. For the synthesized and discussed block copolymers, the subscripts denote the weight fractions in percentage and the superscript the corresponding molecular weight of the block copolymer in kg·mol^−1^.

### 2.3. Block Copolymer Synthesis

As previously reported [47], in a typical sequential anionic polymerization for the formation of PS-*b*-PHEMA the required amount of styrene was dissolved in THF in an ampule equipped with a stirring bar at −72 °C followed by quick addition of *s*-Buli (1.4 M solution in hexane). The solution was stirred for 2 h to ensure complete conversion of styrene. A sample of the yellowish solution was taken and treated with degassed methanol for size-exclusion chromatography (SEC) measurements. 2 eq. of DPE were added and the immediately deep red solution was stirred for additional 30 min. Prechilled HEMA-TMS, which was diluted with THF, was added to the ampule containing the endcapped PS macro anions. The reaction immediately turned colorless. After 2 h of reaction time, the polymerization was terminated by the addition of degassed methanol. For hydroxyl-group deprotection of PHEMA-TMS, concentrated hydrochloric acid was added to the ampule, followed by stirring at room temperature for 2 h. The deprotected block copolymer was precipitated in water, filtrated and dried in vacuo. SEC measurements were carried out using DMF/LiCl as eluent and NMR analysis was carried out using pyridine-*d*_5_ as solvent.

### 2.4. Block Copolymer Modification with Ferrocene Acid

The ferrocene carboxylic acid was synthesized as reported elsewhere [48,49]. In a slightly modified protocol, 40 μL (0.52 mmol; 4 eq.) oxalyl chloride was added to 30 mg (0.13 mmol; 1 eq.) ferrocenic acid in 5 mL dry dichloromethane (DCM) and one drop dimethylformamide (DMF) as catalyst under argon atmosphere at 0 °C. The solution turned deep red and was stirred at room temperature for 1 h. The solvent and excess of oxalyl chloride was removed under reduced pressure and the red residue was diluted in hexane. After filtrating the unreacted ferrocene carboxylic acid, the hexane was removed under vacuum and the red residue was disolved in dry DCM. The purified ferrocenoyl chloride solution was added slowly to a solution of 200 mg PS-*b*-PHEMA (0.27 mmol with respect to available OH groups in the corresponding block copolymer; 2 eq.) in 8 mL dry pyridine at 0 °C. The solution turned orange and was stirred at 0 °C for 3 h. The polymer was precipitated in methanol and dried under reduced pressure. 201 mg (78%) of an orange/brownish powder was obtained. The polymer was characterized using NMR and SEC measurements. The *M_n_* of the final block copolymer was calculated using SEC of the first block segment and the composition of the block copolymer determined by NMR spectroscopy.

### 2.5. Membrane Formation with Ferrocene-Functionalized Block Copolymers

300 mg block copolymer (200 mg PS-*b*-PHEMA and 100 mg ferrocene-functionalized block copolymer) and 2 mg CuCl_2_ (0.1 wt %) were dissolved in a mixture of THF, DMF and dioxane (DOX) (788/394/394 mg i.e., 2:1:1 wt %) resulting in a yellowish 16 wt % casting solution. The solution was casted on a THF-soaked non-woven polymer support with a doctor blade featuring a blade gap of 200 μm. After a certain evaporation time—typically between 10 s and 30 s—the polymer film was immersed into deionized water at room temperature. The polymer films were stored in deionized water prior to use for characterization by microscopy, cyclic voltammetry, or for water flux measurements. For SEM measurements, a small sample of the dried membrane was taken, which was dried at room temperature, followed by additional drying at 40 °C under reduced pressure.

## 3. Results

### 3.1. Polymer Synthesis and Characterization

**Figure 1:**
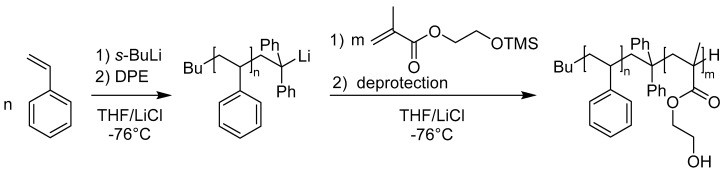
Sequential anionic polymerization of styrene and trimethylsilyl-protected 2-hydroxyethyl methacrylate leading to polystyrene-*block*-poly(2-hydroxyethylmethacrylate) (PS-*b*-PHEMA) block copolymers.

The amphiphilic block copolymers polystyrene-*block*-poly(2-hydroxyethyl methacrylate) (PS-*b*-PHEMA) were synthesized by sequential anionic polymerization of styrene and trimethylsilyl-protected HEMA (HEMA-TMS) as described previously [47]. The deprotection of the PHEMA-TMS was achieved by the addition of HCl to the block copolymer/THF solution (see Experimental Section). The synthetic route leading to PS-*b*-PHEMA is depicted in Figure 1.

The molar masses of the corresponding PS block segement were analyzed by SEC measurements in THF calibrated with PS as standards. The molar mass of the corresponding PS-*b*-PHEMA block copolymers was calculated based on the SEC results of the first block segment followed by calculations based on the content derived by ^1^H NMR spectroscopy measurements. The dispersity index, *Đ*, of the block copolymers was determind by SEC measurements in DMF/LiCl calibrated with PMMA as standards. The characterics as determined by SEC and ^1^H NMR measurements of the investigated block copolymers are compiled in Table 1. An exemplary molecular weight distribution measured in DMF/LiCl and corresponding ^1^H NMR spectrum is shown in the Supporting Information (Figure S1, Supplementary Materials). It should be mentioned that the obtained molar masses by SEC in DMF/LiCl differ from the calculated molar masses compiled in Table 1. The functionalized polymer could not be measured by SEC in THF due to solubility issues. For denotation of the block copolymers—as for instance PS_75_-*b*-PHEMA_25_^80^—the subscripts refer to the weight fraction of the corresponding block segment and the superscript refers to the total molecular weight in kg·mol^−1^.

Block copolymers were successfully synthesized with molecular weights of up to 112 kg·mol^−1^ and a PHEMA content between 13.0 and 21.3 mol %. The low values for the dispersity index, *Đ*, indicate the excellent control over the anionic polymerization protocol. The thermal properties of PS-*b*-PHEMA were investigated by performing DSC measurements (Figure S3, Supplementary Material). The typical transition temperatures for PS is 107 °C [50] and for PHEMA 124 °C, respectively. The high number of hydroxyl-groups in the synthesized block copolymer can be exploited for further functionalization strategies in order to achieve new functional moieties as part of the block copolymer architecture. This opens the path for a lot of functionalization opportunities to tailor the properties of the PS-*b*-PHEMA block copolymers, which will be described in the ensuing section.

### 3.2. Functionalization of PHEMA-Containing Block Copolymers with Redox-Responsive Moieties

**Figure 2:**
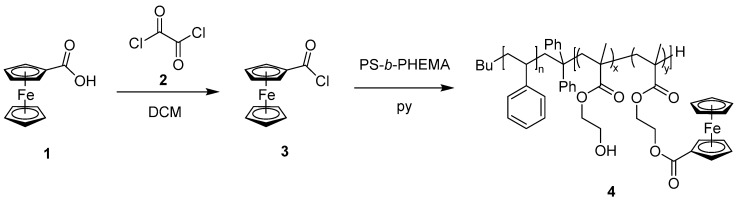
One pot synthesis of the functionalization of PS-*b*-PHEMA with oxalyl chloride **2** activated ferrocene carboxylic acid **1** leading to the novel block copolymer PS-*b*-(PHEMA–*co*–PFcMA) **4**.

The hydroxyl-groups of the amphiphilic block copolymer PS-*b*-PHEMA were exploited for functionalization with the redox-responsive ferrocene carboxylic acid. The postmodification route for the herein investigated block copolymers was based on an established protocol by Zhu et al. for OH groups in a one-pot synthesis [51]. The chemical strategy for postmodification is shown in Figure 2. For this purpose, the ferrocene carboxylic acid **1** was activated using oxalyl chloride **2** and the reaction was performed in DCM and pyridine. This lead to the highly reactive ferrocene carbonyl chloride **3**. The nucleophilic hydroxyl groups readily react with the ferrocene acid chloride leading to PS-*b*-(PHEMA–*co*–PFcMA) **4**. After functionalization the second block segment consists statistically of PHEMA and PFcMA.

**Figure 3:**
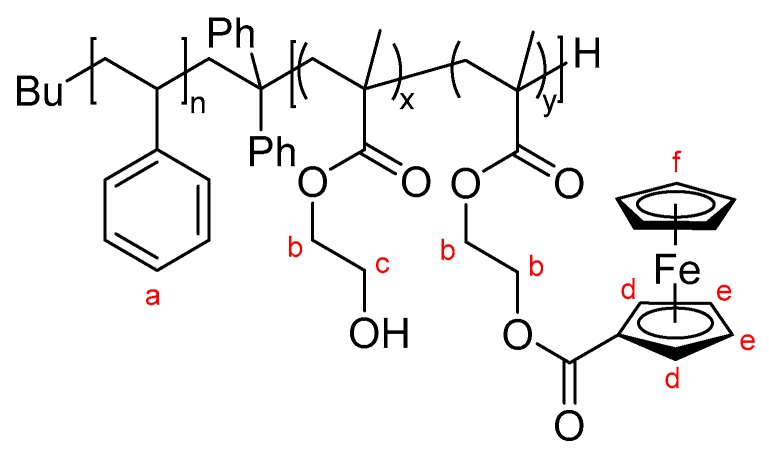

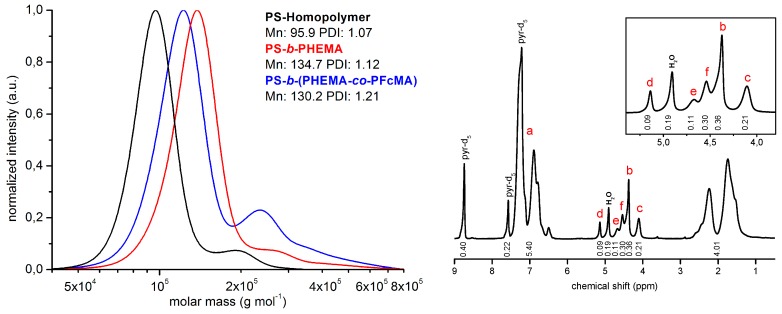
SEC in DMF/LiCl of the first block segment, the block copolymer PS-*b*-PHEMA and the ferrocene-functionalized block copolymer and ^1^H NMR in pyridine-*d*_5_ spectra of functionalized PS_60_-*b*- (PHEMA_13_–*co*–PFcMA_27_)^123^.

Analysis of the obtained functional block copolymers were carried out by SEC in DMF/LiCl and ^1^H NMR spectroscopy in pyridine-*d*_5_. An exemplary molecular weight distribution measured in DMF/LiCl of the ferrocene-functionalized block copolymer PS_60_-*b*- (PHEMA_13_–*co*–PFcMA_27_)^123^ and the corresponding ^1^H NMR spectrum is given in Figure 3. It should be mentioned that the obtained molar masses by SEC in DMF/LiCl differ from the calculated molar masses compiled in Table 2. The functionalized polymer could not be measured by SEC in THF. The degree of functionalization (DF) was calculated via ^1^H NMR measurements. Upon functionalization with the ferrocene carboxylic acid the CH_2_ group of the former alcohol moiety shifted to an higher chemical shift as can be drawn from the inset of Figure 3. The reduction of the integral of this CH_2_ group is characteristic for the amount of ferrocene as part of the block copolymers.

The degree of functionalization (DF) of the performed synthesis, the molar masses of the resulting ferrocene-functionalized block copolymers which were calculated based on the SEC results of the first block segment and via ^1^H NMR spectroscopy measurements and the dispersity indices, *Đ*, obtained by SEC in DMF/LiCl, are given in Table 2.

Noteworthy, the molecular weight distribution of the ferrocene functionalized polymer revealed a smaller molar mass for the main distribution of the modified block copolymers compared to the pristine PS-*b*-PHEMA block copolymers. This is due to the fact that the ferrocene moieties induced a decreased solution capability for the block copolymers in DMF/LiCl. This resulted in a smaller hydrodynamic volume and therefore an apparent smaller molecular weight of the polymer itself was observed [17,52,53,54]. Nevertheless, molar masses can easily be obtained and calculated by ^1^H NMR spectroscopy and the results are additionally compiled in Table 2. It has to be mentioned, that a second less intensive distribution, which is shifted to higher molar masses, could be observed by SEC measurements. This is maybe a result of coupling of polymer chains during the functionalization step or some oxygen residues during the anionic polymerization. Nevertheless, the obtained low polydispersity indices proved the excellent control over anionic synthesis and herein established postmodification route, which is of crucial importance for the porous membrane formation. Additionally, the thermal properties of PS-*b*-PHEMA were investigated by TGA and DSC measurements. TGA measurement of the pristine and ferrocene-modified BCPs were performed up to a temperature of 600 °C. The residue of pure PS-*b*-PHEMA was determined to be 0.5%, whereas the residue of the ferrocene-functionalized block copolymer was determined to be 3.4%. As another hint for successful ferrocene-modification, the residue as obtained after treatment at 600 °C turned out to be magnetic, which is typical for ferrocene-containing polymers after ceramization [54,55]. This finding is in good agreement with theoretical calculations for the iron content of the block copolymers. For comparison, a TGA of PFcMA (Figure S2, Supplementary Materials) is given. While pure PFcMA featured a ceramic yield of iron oxide of 25%, the ceramic yield of a PS–PHEMA featuring 4.4 mol % (12.9 wt %) of FcMA moieties was 3.4%. The theoretical amount of resulting iron oxide for PFcMA is 23.2% and for the ferrocene-functionalized polymer 3.0% which is in good agreement of the measured residue and again proved the justification of ferrocene-modification and NMR analysis of the ferrocene-containing block copolymers. DSC analysis of the block copolymer showed the typical transition temperatures for PS is 106.5 °C and for the statistical second block consisting of PHEMA and PFcMA a transition temperature of 122.6 °C. An exemplary DSC curve of PS_60_-*b*-(PHEMA_13_–*co*–PFcMA_27_)^123^ is given in Figure S3. However, there is no additional glass transition temperature for a PFcMA block segment—which would be expected at about 80 °C [56]—(Figure S3, Supplementary Materials). Compared to the intensity and value of the PHEMA glass transition temperature, the signal is slightly reduced which is assumed to be the result of a statistical composition of PHEMA and PFcMA in the second block segment. In summary, obtained results by NMR spectroscopy, TGA and SEC prove the success of PS-*b*-PHEMA modification with ferrocene moieties. In the next step, structure formation in binary and ternary solvent mixtures is investigated, which is of crucial importance for the intended membrane formation.

**Figure 4:**
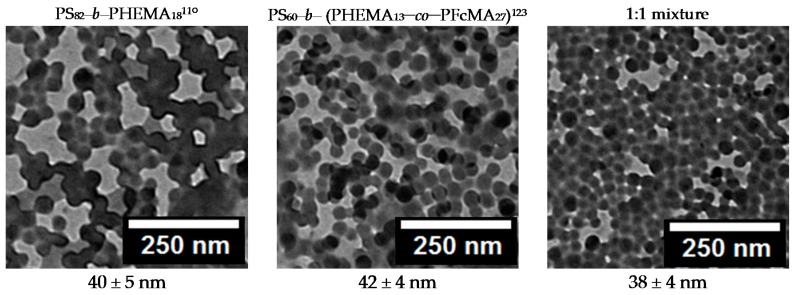
TEM images of block copolymer micelles of PS_82_-*b*-PHEMA_18_^110^, ferrocene functionalized PS_60_-*b*-(PHEMA_13_–*co*–PFcMA_27_)^123^ and a 1:1 wt % mixture of the block copolymers. The used solvent system was THF/DMF/DOX 2:1:1 wt %.

The formation of uniform micelles of the ferrocene-functionalized block copolymers is very important for the self-assembly and non-solvent induced phase separation (SNIPS) process. Therefore, the novel block copolymers were investigated by transmission electron micrsocopy (TEM) from their micellar solution in different solvent mixtures, which are typical for the membrane casting procedure. Exemplarily, a 1 wt % solution of the functionalized block copolymer in a solvent mixture consisting of THF/DMF/DOX (2:1:1 wt %) was prepared and drop-casted on a carbon-coated copper grid. The dried micelles on the carbon-coated copper grid were investigated by TEM measurements (Figure 4). Furthermore, the micellation of polymer mixtures of pure PS-*b*-PHEMA and functionalized block copolymer PS-*b*-(PHEMA–*co*–PFcMA) was investigated. The resulting TEM images are shown in Figure 4.

It can be concluded from these TEM images that the micelles of pure PS-*b*-PHEMA, ferrocene-functionalized block copolymer and of a mixture of pure and functionalized block copolymer reveal uniform spherical micelles featuring diameters of 40 ± 5 nm, 42 ± 4, and 38 ± 4 nm, respectively. Upon functionalization with ferrocene, there is no significant change for the tendency of the block copolymers to form micelles and the size of the micelles was not much changed. Due to the block copolymer composition as well as the used solvent mixture, the corona of the micelles consist of the PS segment whereas the core fo the corona consist of the PHEMA or the PHEMA–*co*–PFcMA segment. These results are a good indication that the ferrocene-modified block copolymers can be used in a similar manner to the pure PS-*b*-PHEMA BCPs without changing the composition of the solvent mixture for the membrane formation by applying the SNIPS process. Moreover, the possibility to use mixtures of functionalized and pure block copolymer should pave the way to easily tailor the amount of redox-responsive functional groups as part of the inner pore walls of the membranes.

### 3.3. Membrane Preparation by Self-Assembly and Non-Solvent-Induced Phase Separation (SNIPS) Process

**Figure 5:**
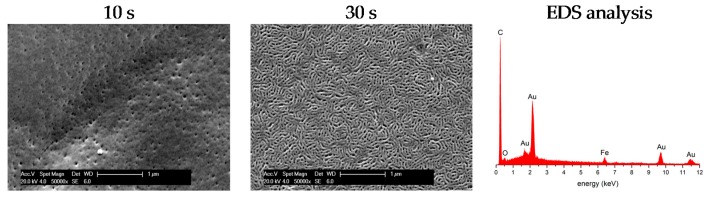
SEM images and EDS anaylsis of block copolymer membranes prepared of a 2:1 wt % mixture of PS_82_-*b*-PHEMA_18_^110^ and PS_60_-*b*-(PHEMA_13_–*co*–PFcMA_27_)^123^ at different evaporation times. The samples were coated with gold prior to SEM investigation.

As described in the introduction, the microphase separation of block copolymers in solvent mixtures can be advantageously used for the preparation of integral asymmetric membranes by SNIPS process [42,43,46,57]. In general, the pore structure formation is tremendously influenced by a variety of parameters such as, e.g., evaporation time, solvent mixture, additives like salts, humidity, temperature, etc. [44,57,58,59]. Recently, the Abetz group elucidated the influence on membrane formation by using scattering methods [58]. In general, the matrix of the final membrane consists of the PS segment, whereas the cylindrical domains and the inner surface of the pores will be formed by the hydrophilic block segment. In the present study, this is the PHEMA and PHEMA–*co*–PFcMA block segment. For the SNIPS process, a 16 wt % block copolymer solution of a mixture of pure and functionalized block copolymer (1:1 or 2:1 wt %) consisting of THF/DMF/DOX (2:1:1 wt %) and 0.1 wt % CuCl_2_ as additive [45,60] was prepared and the viscous solution was casted on top of a non-woven polymer support by a doctor blade with a gap height of 200 μm (see Experimental Section). THF and DOX are better solvents for the PS segment, whereas DMF is better for the PHEMA segment [47]. Upon evaporation mainly THF evaporates leading to an enrichment of the block copolymer on the top surface of the film causing the asymmetric structure of the block copolymer membrane. Hence the high density of block copolymer at the surface of the casted film lead to microphase separation of the block copolymer, which results after precipitation in the ordered and dense morphology of the membrane surface. SEM results for the ferrocene-functionalized block copolymer mixtures of PS_82_-*b*-PHEMA_18_^110^ and PS_60_-*b*-(PHEMA_13_–*co*–PFcMA_27_)^123^ (2:1 wt %) are given in Figure 5. Furthermore, the evaporation time during the SNIPS process of 1:1 mixtures of ferrocene-functionalized block copolymer and PS-*b*-PHEMA was investigated (also see Supplementary Materials, Figure S4).

As shown previously, the SNIPS process lead to the formation of integral asymmetric membranes obtained by the block copolymer PS-*b*-PHEMA featuring open pores in a perfect hexagonal lattice [47]. Compared to these findings, the membranes prepared from solutions of the ferrocene-functionalized block copolymers form dense surface morphologies with less ordered pores. Moreover, a lying cylinder-like topology depending on the increasing evaporation time was observed. Especially the membrane, with an evaporation time of 30 s, has a highly porous top surface structure with similar size distributions of the porous areas (Figure 5, middle). Additionally, SEM and obtained energy-dispersive X-ray spectra confirmed the presence of iron inside the porous membrane and therefore the presence of ferrocene-moieties (Figure 5, right). In Figure S4 the membrane obatined from the casted 1:1 polymer mixture was investigated by SEM. It can be concluded from these images that with increasing evaporation time, the pore density and therefore the porosity of these membranes is reduced on the surface. Upon an evaporation time of 30 s these membranes showed no lying cylinder-like topography as the membranes discussed before. Nevertheless, an open-porous structure could be observed without cracks or additional defect structures for every obtained membrane, which is crucial for the intended redox-induced filtration investigations as described in the ensuing section.

### 3.4. Stimuli-Responsiveness of the Membranes and Water Flux Measurements

**Figure 6:**
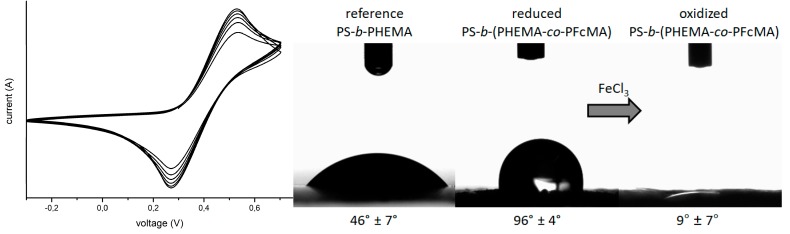
**Left**: Cyclic voltammogram for a PS_60_-*b*-(PHEMA_13_–*co*–PFcMA_27_)^123^ as thin film. Shown are five cycles at a rate of 20 mV·s^−1^; **Right**: Contact angles obtained for a reference membrane consisting of PS-*b*-PHEMA and of the ferrocene functioanlized membranes in a reduced and oxidized state.

The ferrocene-moieties of the functionalized block copolymer can be electronically or chemically addressed to change the oxidation state of the iron [23,29]. The stimuli-responsive behaviour of the ferrocene-functionalized block copolymer was analyzed by cyclovoltammetry. As the membrane does not feature an intrinsic conductivity, the initial CV measurement was carried out from a thin film on the surface of a glassy carbon electrode. This measurement should give a first proof for the acccessibility of the ferrocene moieties at the top surface of the block copolymer membrane. For this experiment, 0.1 M tetrabutylammonium tetrafluoroborate (TBAF_4_) solution in degassed acetonitrile as electrolyte was chosen, which is a non-solvent for the block copolymer. The obtained cyclovoltamogram is given in Figure 6. The redox-responsive behaviour of the ferrocene-functionalized block copolymer indicate that the system can be oxidized and reduced in a reversible manner (here shown for 5 cycles). In the next step, the membrane was chemically oxidized by using a 0.2 wt % solution of iron(III) chloride in water for 24 h. After rinsing in water in order to remove excess of oxidizing agent, the membranes were dried for contact angle (CA) measurements. Due to the oxidation of ferrocene to ferrocenium, the color of the former light brown membrane intensified to brown. The CA study of a PS-*b*-PHEMA reference membrane, a reduced and an oxidized ferrocene-functionalized membrane was performed in a climate chamber using droplets of deionized water. The corresponding CAs of the samples were measured at various positions of the membrane (Figure 6).

As a result, the ferrocene-functionalized membrane revealed higher CA values (96° ± 4°) than the PS-*b*-PHEMA reference membrane (46° ± 7°). This is caused by the more hydrophobic character of the ferrocene moieties. Upon oxidation, the membrane increased in polarity which is nicely reflected by the obtained CA results of 9° ± 7°. This finding clearly proved the tremendous change of polarity for the ferrocene-modified membrane by convenient oxidation with iron(III) chloride.

The herein obtained novel redox-responsive membranes based on modified PS-*b*-PHEMA are investigated by water flux measurements in order to elucidate the effect of oxidation on the filtration performance of the membranes. The experiments were carried out with a stirred flow cell (Merck Amicon 8010, 2.5 cm membrane diameter) and a constant pressure of 1.0 bar. As previously reported, [47] pure PS-*b*-PHEMA based membranes revealed high water fluxes up to 2700 L m^−2^·bar^−1^·h^−1^. The obtained water flux for the membrane in a reduced ferrocene state was determined to be 663 L·m^−2^ bar^−1^·h^−1^, whereas in the oxidized state the obtained water flux was 34 L m^−2^·bar^−1^·h^−1^. The water flux of the ferrocene-functionalized membrane turned out to be significantely lower than the pure PS-*b*-PHEMA membrane. We assume, that this is due to (i) the less polar character of the ferrocene moieties at the inner pore surface and (ii) because of the more dense structure of the membrane. Upon oxidation of the membrane the water flux was tremendously reduced (up to 90%). This can be explained similar to other stimuli-responsive membranes, when charges were introduced inside the pores [40,42]. The oxidized ferrocene moieties act in a similar manner as a polyelectrolyte: the repulsion of charges of the statistical PHEMA–*co*–PFcMA segment leads to a polymer chain stretching, which reduces the hydrodynamic pore diameter and thus the water flux. This result indicated that ferrocene-functionalized membranes can be used for redox-switching the water flux. Most importantly, the overall top-surface structure of these novel membranes did not change after water flux measurements or after the oxidation of the ferrocene moieties. This was confirmed by SEM measurements (Supplementary Materials, Figures S5 and S6) again proving that the switching capability of the metallopolymer-based polyelectrolyte is the reason for the influence on the water flux. Furthermore, due to the positive charge inside of the pores/membrane this system can be used for ion separation techniques, which will be investigated in the near future. Recently, ferrocene-containing polymer were shown to selective bind anions [61]. This could be advantageously used for selective purification processes of porous metallopolmyer architectures.

## 4. Conclusions

This paper presented the fabrication of redox-responsive integral asymmetric membranes, which were obtained by the functionalization of the amphiphilic block copolymer polystyrene-*block*-poly(2-hydroxyethyl methacrylate) (PS-*b*-PHEMA) with redox-responsive moieties based on ferrocene. The polymers were successfully functionalized leading to polystyrene-*block*-(poly(2-hydroxyethyl methacrylate)–*co*–poly((2-methacryloyloxy)ethyl ferrocene carboxylate)) (PS-*b*-(PHEMA–*co*–PFcMA)) with a PFcMA content of up to 66% and molar masses up to 123 kg·mol^−1^. By taking advantage of the self-assembly of the block copolymers by application of the non-solvent induced phase separation (SNIPS) process, integral asymmetric membranes were obtained. The matrix of the membrane is formed by the PS segment whereas the cylindrical domains and the inner surface of the pores are formed by the PHEMA and PFcMA domains. The porous topography was shown via scanning electron microscopy (SEM) and the redox-responsive behavior was demonstrated using cyclic voltammetry. The water flux of the ferrocene functionalized membrane in the oxidized state was with 34 L h^−1^·m^−2^·bar^−1^ significantly lower than in the reduced state with 663 L h^−1^ m^−2^ bar^−1^ allowing these novel membranes to switch the water flux upon oxidation/reduction. The herein reported new redox-responsive membranes could enable the selective separation of ions in an aqueous system upon filtration due to the presence of positive charges at the ferrocene moieties.

## Figures and Tables

**Table 1:** Characterization of synthesized block copolymers PS-*b*-PHEMA obtained by SEC measurements and ^1^H NMR spectroscopy.

Block Copolymer	*M*_n_^a^	*Đ* ^b^	x_HEMA_^c^
PS_75_-*b*-PHEMA_25_^80^	80	1.14	21.3
PS_84_-*b*-PHEMA_16_^100^	100	1.07	13.0
PS_82_-*b*-PHEMA_18_^106^	106	1.12	14.9
PS_82_-*b*-PHEMA_18_^110^	110	1.18	14.5
PS_75_-*b*-PHEMA_25_^112^	112	1.11	20.6

^a^ Molar masses in kg·mol^−1^ calculated by SEC analysis of the PS segment (PS standards, Tetrahydrofuran (THF) and ^1^H NMR data of the block copolymer; ^b^ *Đ* values correspond to the SEC analysis of the block copolymers in DMF/LiCl; ^c^ Content of HEMA determined by ^1^H NMR.

**Table 2:** Characterization of ferrocene-functionalized block copolymers PS-*b*- (PHEMA–*co*–PFcMA) via SEC measurements and ^1^H NMR spectroscopy.

Block Copolymer	*M*_n_^a^	*Đ* ^b^	DF ^c^
PS_72_-*b*- (PHEMA_17_–*co*–PFcMA_11_)^112^	112	1.41	19
PS_61_-*b*- (PHEMA_10_–*co*–PFcMA_29_)^113^	113	1.77	53
PS_77_-*b*- (PHEMA_13_–*co*–PFcMA_11_)^115^	115	1.59	30
PS_72_-*b*- (PHEMA_5_–*co*–PFcMA_23_)^117^	117	1.76	66
PS_78_-*b*- (PHEMA_14_–*co*–PFcMA_17_)^121^	121	1.37	20
PS_60_-*b*- (PHEMA_13_–*co*–PFcMA_27_)^123^	123	1.21	43
PS_64_-*b*- (PHEMA_12_–*co*–PFcMA_24_)^132^	132	1.66	44

^a^ Molar masses in kg·mol^−1^ calculated by SEC analysis of the PS segment (PS standards, THF) and ^1^H NMR data of the functionalized block copolymer; ^b^ *Đ* values correspond to the SEC analysis of the functionalized block copolymers in DMF/LiCl; ^c^ Degree of Functionalization calculated via ^1^H NMR.

## Supplementary Materials

The following are available online at www.mdpi.com/2073-4360/9/10/491/s1. Figure S1: SEC and ^1^H NMR spectrum for polymer PS_82_-b-PHEMA_18_^105^, Figure S2: TGA of PS_82_-*b*-PHEMA_18_^106^ and PS_60_-*b*-(PHEMA_13_-*co*-PFcMA_27_)^123^ and homopolymers, Figure S3: DSC for block copolymers, Figure S4: Additional SEM images of ferrocene-functionalized block copolymer membranes, Figure S5: Additional SEM image of ferrocene-functionalized block copolymer membrane after water-flux measurement, Figure S6: Additional SEM image of ferrocene-functionalized block copolymer membrane after oxidation.
