# Supplementary material for: Ferrocene-Modified Block Copolymers for the Preparation of Smart Porous Membranes
Source: Polymers (Basel). 2017 Oct 8;9(10):491. doi: 10.3390/polym9100491 (PMC6418580; doi:10.3390/polym9100491)
Supplement: Supplementary file 1 [file polymers-09-00491-s001.docx]

**Supporting Information**


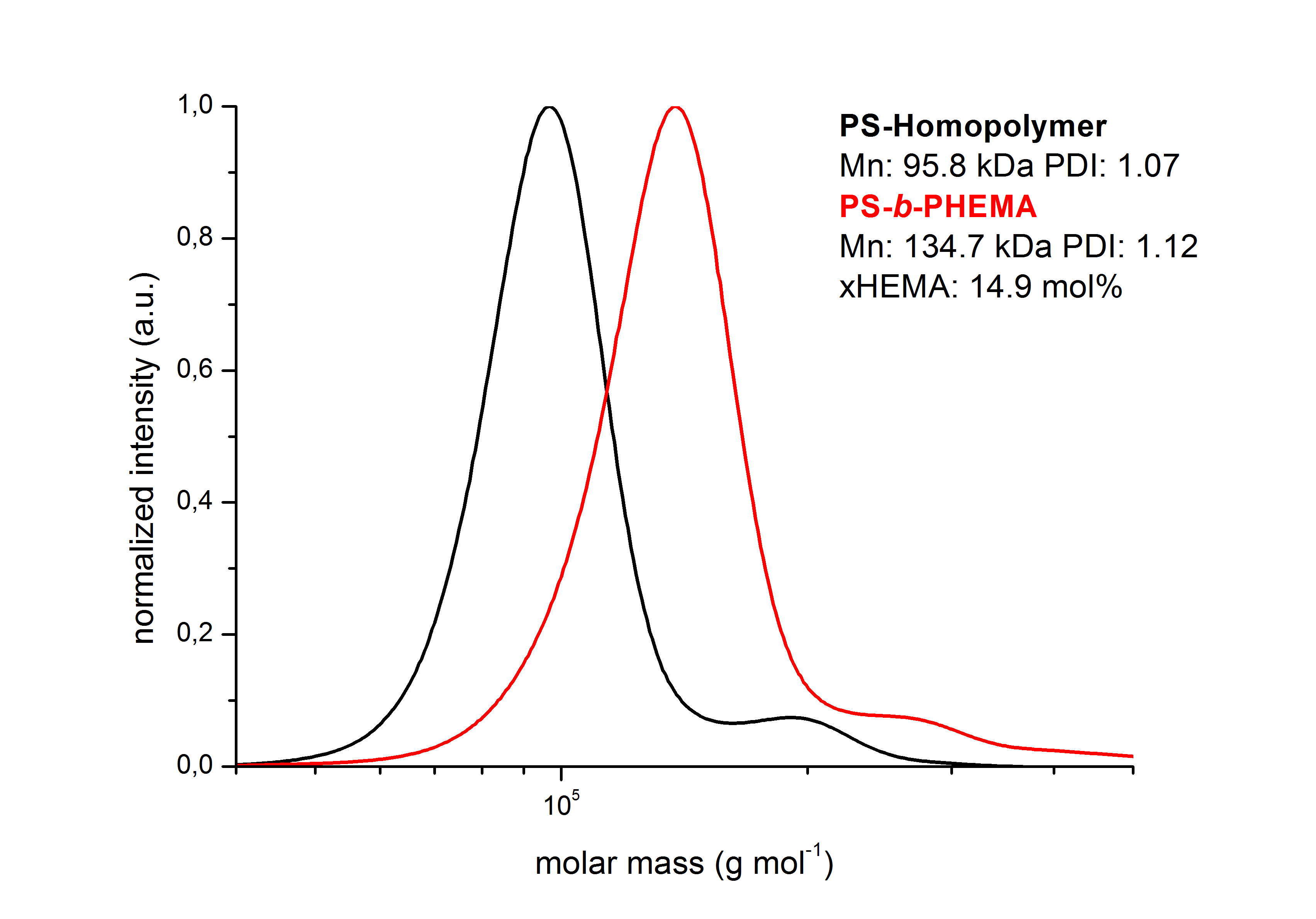

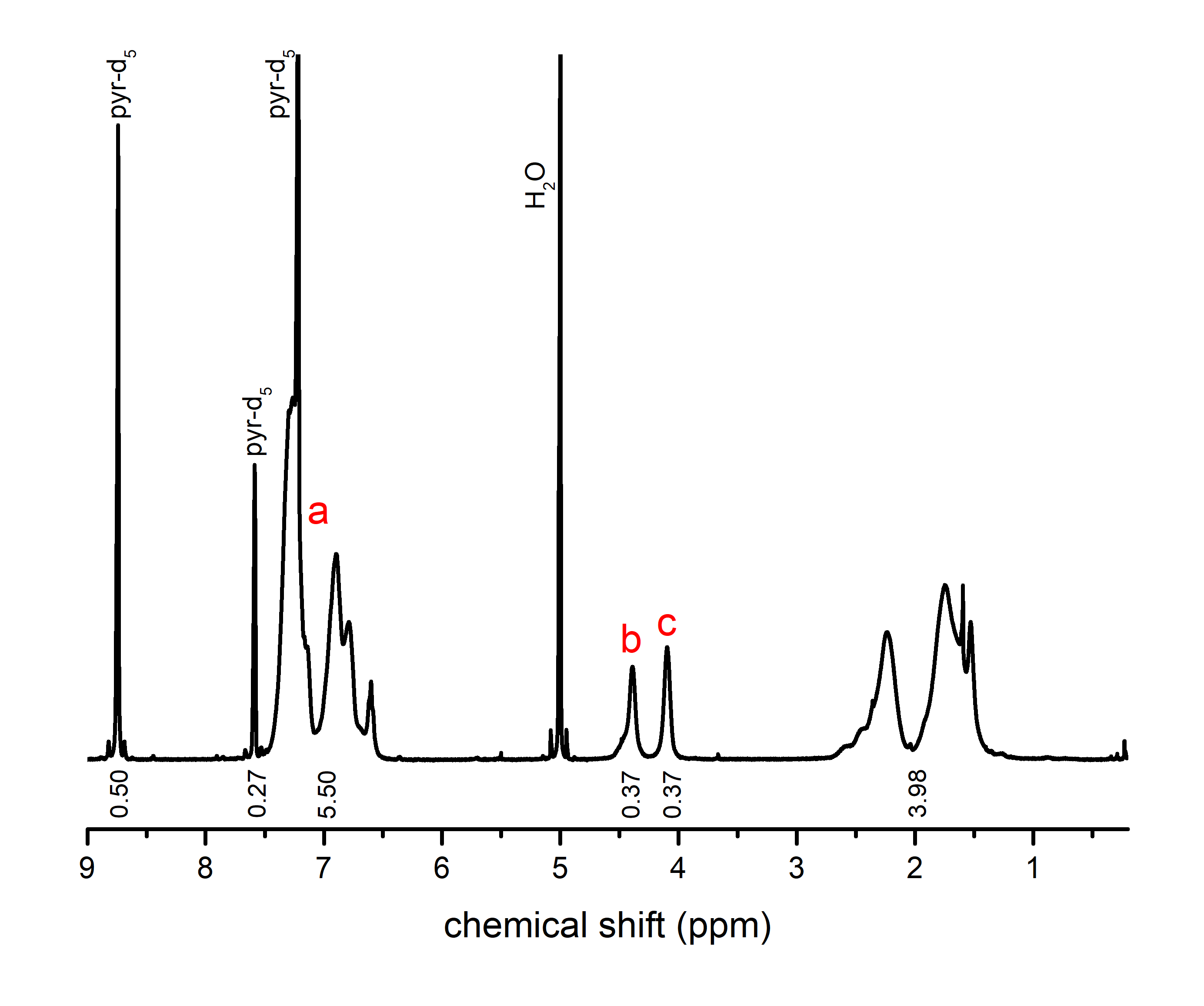

**Figure S1.** SEC in DMF/LiCl and ^1^H NMR in pyridine-d_5_ spectra of PS_82_-b-PHEMA_18_^105^.

**
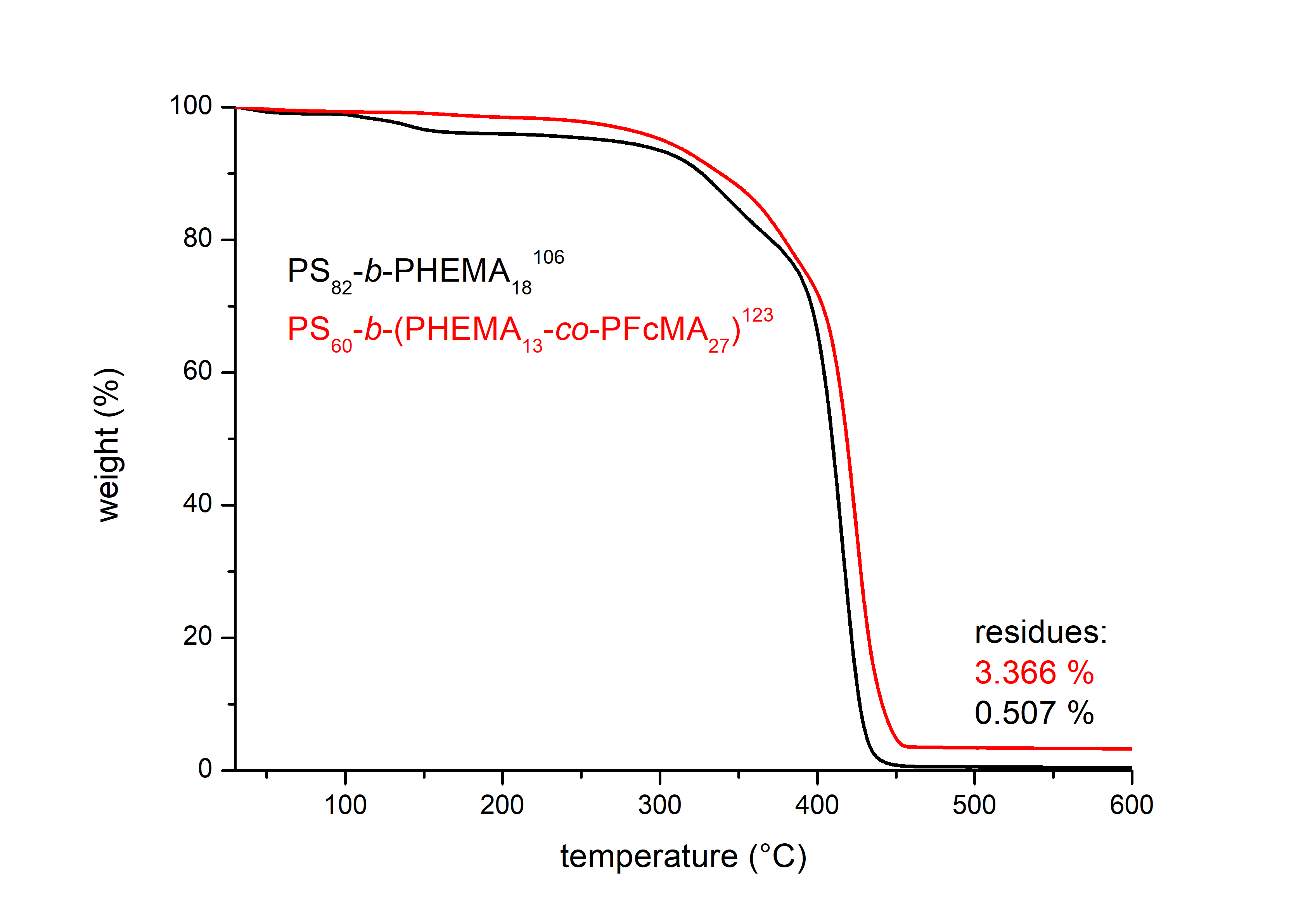

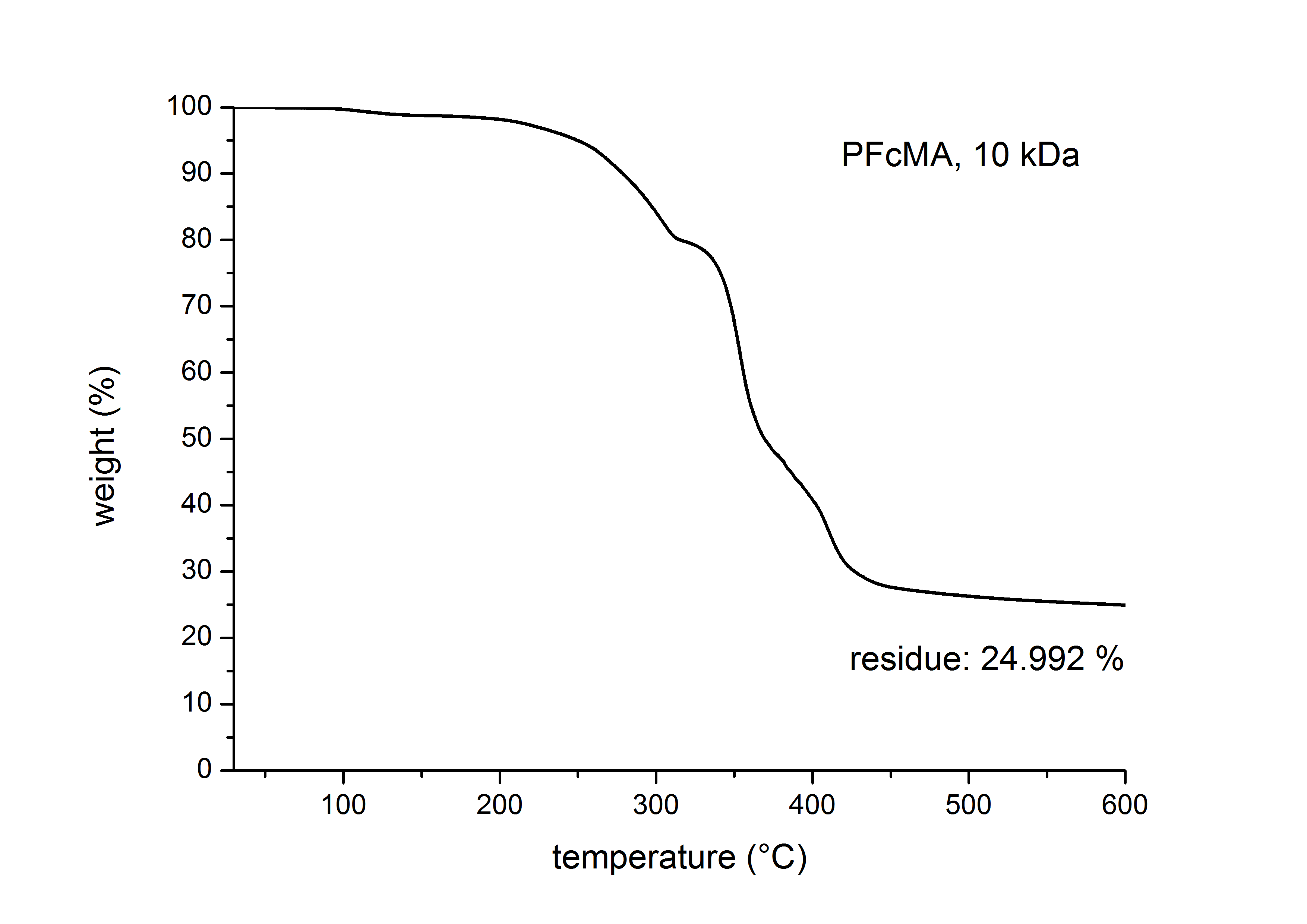
**

**Figure S2.** Left: TGA of PS_82_-*b*-PHEMA_18_^106^ (black) and of PS_60_-*b*-(PHEMA_13_-*co*-PFcMA_27_)^123^ (red). Right: TGA of PFcMA homopolymer with a molar mass of 10 kg mol^-1^. The TGA was performed starting from room temperature ending at 600 °C with a heating rate of 10 K min^-1^.


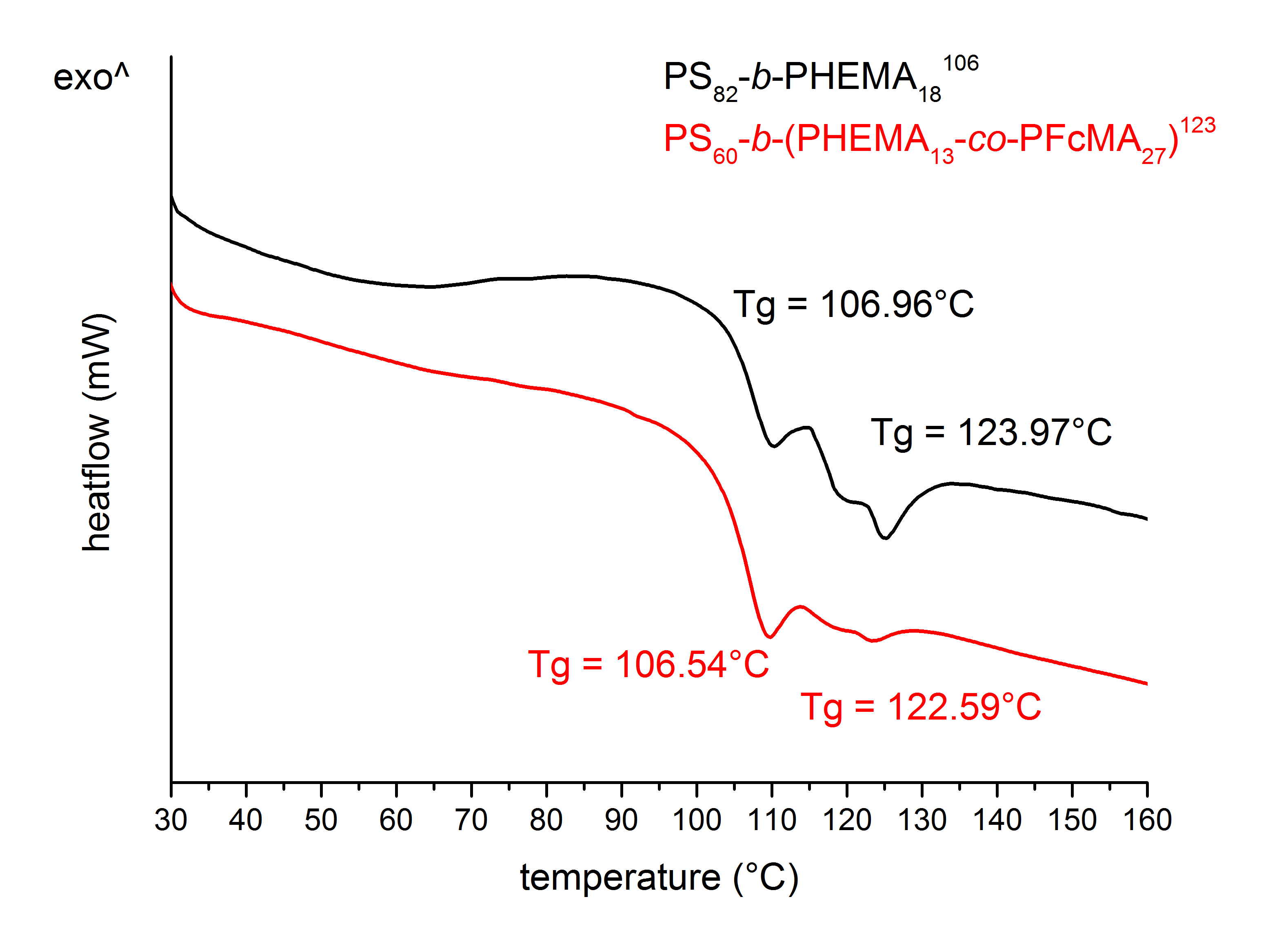

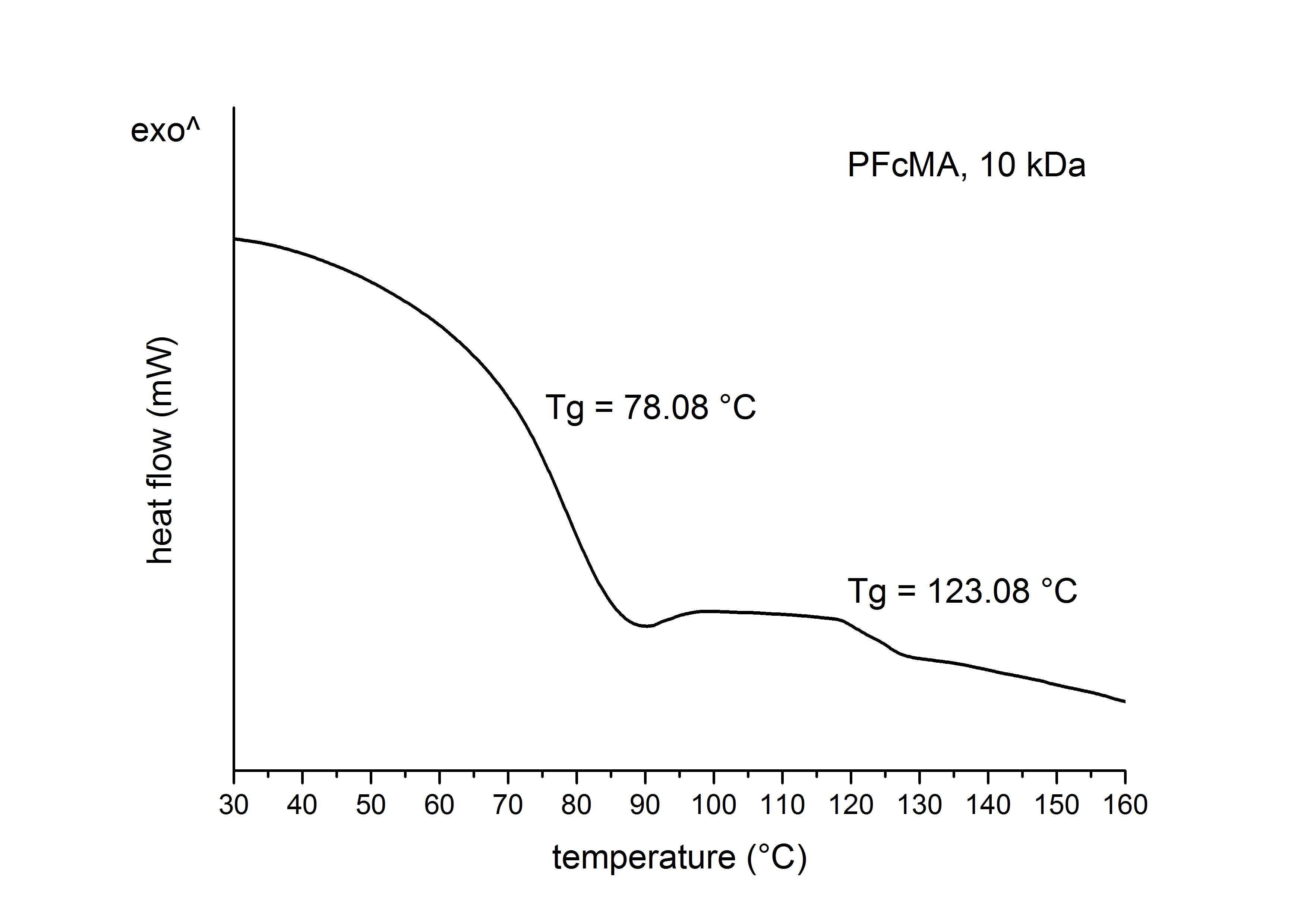


**Figure S3.** Left: Thermogram of precursor PS_82_-*b*-PHEMA_18_^106^ and of ferrocene functionalized PS_60_-*b*-(PHEMA_13_-*co*-PFcMA_27_)^123^. Right: Thermogram of PFcMA homopolymer with a molar mass of 10 kg mol^-1^. The DSC was performed starting from room temperature ending at 180°C with a heating rate of 10 K min^-1^.


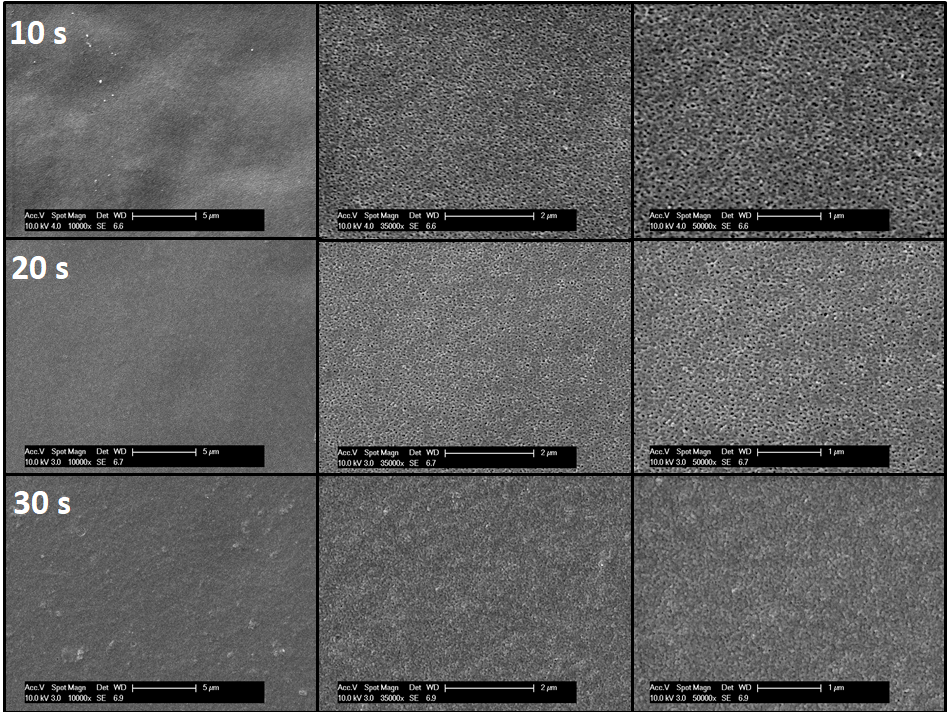


**Figure S4.** SEM images of ferrocene-functionalized block copolymer membranes prepared of a 1:1 wt % mixture of PS_75_-*b*-PHEMA_25_^112^ and PS_64_-*b*-(PHEMA_12_-*co*-PFcMA_24_)^132^ at different evaporation times and different magnitudes. The sample was coated with gold prior SEM investigation.





**Figure S5.** SEM image of oxidized block copolymer membrane prepared of a 2:1 wt % mixture of PS_82_-b-PHEMA_18_^110^ and PS_60_-*b*-(PHEMA_13_-co-PFcMA_27_)^123^ at an evaporation time of 30s after water flux measurement. The membrane was oxidized using a 0.2 wt % FeCl_3_ solution in water. The sample was coated with gold prior SEM investigation.


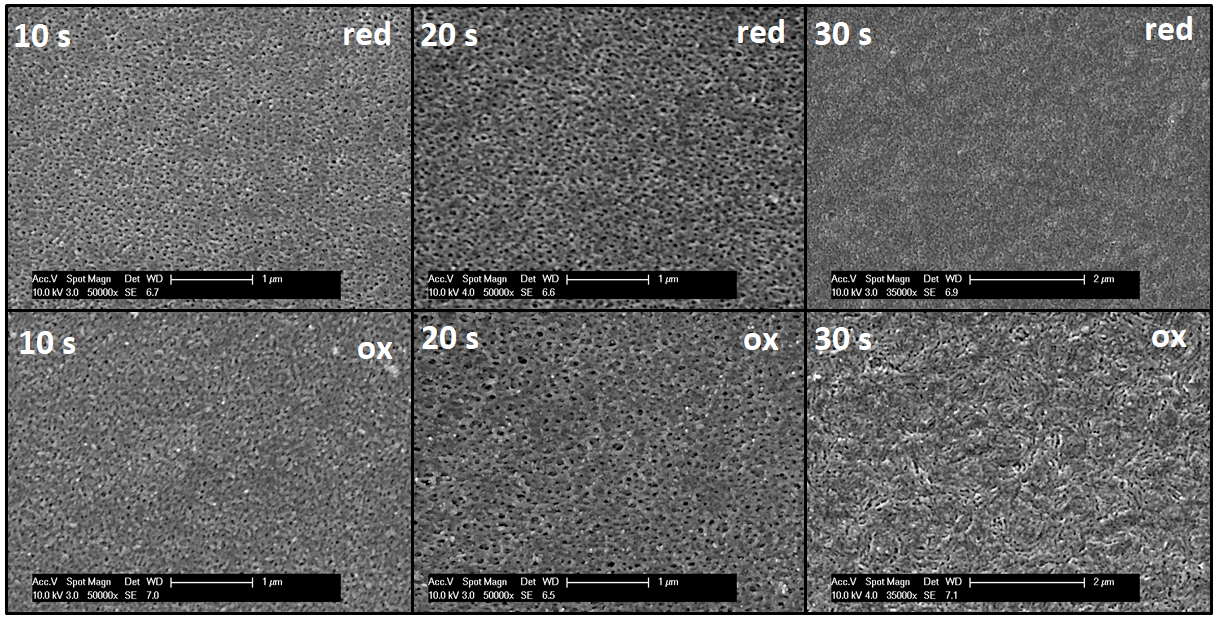


**Figure S6.** SEM image of oxidized block copolymer membrane prepared of a 1:1 wt% mixture of PS_75_-*b*-PHEMA_25_^112^ and PS_64_-*b*-(PHEMA_12_-*co*-PFcMA_24_) at different evaporation times before and after oxidation. The membrane was oxidized using a 0.2 wt % FeCl_3_ solution in water. The sample was coated with gold prior SEM investigation.
